# Propofol infusions using a human target controlled infusion (TCI) pump in chimpanzees (*Pan troglodytes*)

**DOI:** 10.1038/s41598-020-79914-7

**Published:** 2021-01-13

**Authors:** T. Miyabe-Nishiwaki, A. Kaneko, A. Yamanaka, N. Maeda, J. Suzuki, M. Tomonaga, T. Matsuzawa, K. Muta, R. Nishimura, I. Yajima, D. J. Eleveld, A. R. Absalom, K. Masui

**Affiliations:** 1 Primate Research Institute, Kyoto University, Inuyama, Aichi Japan; 2 Chubu Gakuin University, Seki, Gifu, Japan; 3 Laboratory of Veterinary Surgery, Graduate School of Agricultural and Life Sciences, The University of Tokyo, Bunkyo-ku, Tokyo, Japan; 4 Department of Pharmacy, National Defense Medical College Hospital, Tokorozawa, Saitama Japan; 5 University Medical Center Groningen, Groningen, Netherlands; 6 Department of Anesthesiology, Showa University School of Medicine, Shinagawa-ku, Tokyo, Japan

**Keywords:** Biological techniques, Zoology

## Abstract

Chimpanzees are genetically and physiologically similar to humans. Several pharmacokinetic models of propofol are available and target controlled infusion (TCI) of propofol is established in humans, but not in chimpanzees. The purpose of this study was to investigate if human pharmacokinetic models can accurately predict propofol plasma concentration (Cp) in chimpanzees and if it is feasible to perform TCI in chimpanzees. Ten chimpanzees were anaesthetized for regular veterinary examinations. Propofol was used as an induction or maintenance agent. Blood samples were collected from a catheter in a cephalic vein at 3–7 time points between 1 and 100 min following the propofol bolus and/or infusion in five chimpanzees, or TCI in six chimpanzees. Cp was measured using high-performance liquid chromatography. The Marsh, Schnider and Eleveld human pharmacokinetic models were used to predict Cp for each case and we examined the predictive performances of these models using the Varvel criteria *Median PE* and *Median APE*. *Median PE* and *Median APE* for Marsh, Schnider and Eleveld models were within or close to the acceptable range. A human TCI pump was successfully maintained propofol Cp during general anesthesia in six chimpanzees. Human propofol pharmacokinetic models and TCI pumps can be applied in chimpanzees.

## Introduction

Chimpanzees (*Pan troglodytes*) are the non-human primates which are physiologically and genetically most similar to humans, and their behavior and cognitive abilities have been intensively studied^1, 2^. They are endangered in the wild and captive chimpanzees live in many research institutions, zoological institutions and sanctuaries. In Japan, there were 302 chimpanzees in 48 institutions as of Dec. 20th 2020 (Great Ape Information Network, https://shigen.nig.ac.jp/gain/). Although they are no longer used in invasive studies, anesthesia is essential for regular and occasional veterinary examinations and treatments. However, published information on anesthetic techniques in chimpanzees are limited. Most published articles consider combinations of intramuscular anesthetics/sedatives/analgesics^3–7^.

For long duration anesthesia, inhalation anesthetics such as isoflurane and sevoflurane are mainly used. The advantages of inhalation anesthetics are that anesthetic machines are easy to use and it is possible to measure anesthetic concentration in exhaled breath in real time. However, there are disadvantages of inhalation anesthetics including the risks of environmental pollution and exposure of personnel to inhalational anesthetics, and furthermore anesthetic circuits and vaporizers are not always available. In addition, chimpanzees sometimes seem to have nausea and vomiting, and they often cough after extubation (anecdotal observations in Kyoto University Primate Research Institute, KUPRI).

In human medicine, total intravenous anesthesia using propofol and analgesics has been developed and is widely used^8^. However, it is not possible to measure blood concentration of propofol in real time as it is for inhalation anesthetics. To compensate for the inability of real time measurement, it is possible to calculate and predict blood or brain concentration of propofol when pharmacokinetic parameters are available in the species. In human medicine, target control infusion (TCI) systems are used, which control blood or effect site (brain) concentrations based on a pharmacokinetic model^8^. TCI syringe pumps incorporating pharmacokinetic models in human are commercially available and when using a TCI pump, an anesthesiologist can set and adjust the target plasma or effect-site concentration depending on the patient needs and the pump automatically controls the infusion rate so that the blood/effect-site concentration maintains at the target concentration. There are several well-known human pharmacokinetic models for propofol, including the Marsh^9^, Schnider^10^ and Eleveld models^11, 12^. Briefly, the Marsh model was developed based on an evaluation of the pharmacokinetics of propofol in 18 patients. The rate constants are fixed whereas compartment volumes and clearances scale linearly weight. The Schnider model was developed based on data from 24 healthy volunteers. It has fixed values for compartment volumes V_1_, V_3_, rate constant k_13_ and k_31_, whereas V_2_, k_12_ and k_31_, are adjusted for age. The metabolic rate constant (k_10_) is adjusted according to total weight, lean body mass and height. The Eleveld models were based on data from multiple institutions; more than 15,000 observations from more than 1000 individuals with wide age and weight ranges. Eleveld models uses allometric scaling and the covariates in Eleveld models include age, weight, height, and sex and post-menstrual age.

In Japan, only TCI pumps incorporating the Marsh model are commercially available. In European countries, so-called “open-TCI pumps” are available, which are programmed with models for propofol (Marsh and Schnider) as well as models for remifentanil and in some cases for alfentanil, ketamine and sufentanil. As propofol is associated with a rapid, clear-headed recovery in humans, which is thought to result in better the early postoperative patient well-being compared to inhalation anesthesia^13, 14^. The incidence of nausea and vomiting is significantly lower after propofol intravenous anesthesia than after inhalation anesthesia in humans^13–17^. Moreover, the incidence of agitation is also lower following propofol anesthesia compared to sevoflurane anesthesia^16^. If it is possible to use human propofol TCI pumps in chimpanzees, it may facilitate the titration of propofol administration for general anesthesia and improve the quality of anesthesia as well as post anesthetic recovery.

The objectives of this study were to investigate (1) if any of the human pharmacokinetic models could accurately predict chimpanzee plasma propofol concentration, and (2) if propofol TCI in chimpanzee is feasible using human TCI pumps.

## Results

Ten chimpanzees (seven females and three males) were anesthetized for regular veterinary examination from 2014 to 2018. The demographic data is listed along with anesthetic protocols in Tables 1 and 2. A chimpanzee named Pendesa had two occasions for regular veterinary examinations during the period. Anesthesia in all chimpanzees were smooth and uneventful except for apnea that was seen in Pendesa. All scheduled regular veterinary examinations including X-ray, blood samplings, tuberculin skin test, with or without dental examinations and CT and/or MRI scan were performed without problems and the chimpanzees recovered well.

**Table 1:** Propofol administration in chimpanzees (2014–2016). MMK: Medetomidine 0.012 mg/kg+Midazolam 0.12 mg/kg + Ketamine 3.5 mg/kg

Name GAIN#	Age (at anesthesia)	Sex	Body weight (kg)	Pre-med	Induction	Maintenance
Pendesa^18, 19^ GAIN# 0095	37	♀	50.8	Diazepam syrup 8 mL po + honey 15 g	MMK Training and Squeezing cage	**Ketamine** (10mg/mL): 6 mL/h CRI → 0.5 mL iv bolus → 10 mL/h CRI **Propofol**: 5 mL iv blous (for intubation) **Sevoflurane: 2**–**3.5%**
Mari GAIN# 0274	39	♀	52.4	Midazolam 5A (50 mg) + glucose Spat out	MMK Telinject	**Propofol**: 50 mg iv bolus → 16 mg iv bolus → 12 mg iv bolus → 5 mg iv bolus (10 min interval) → 30 mL/h CRI → 20 mL/h CRI **Sevoflurane: 3–4**%
Pal GAIN# 0611	15	♀	52.3	Midazolam 5A (50 mg) + glucose Spat out	MMK Telinject	**Propofol**: 55 mg iv bolus → 143 mg Iv bolus(300 mL/h) **Sevoflurane: 2.5–3.5%**
Reo^20^ GAIN# 0439	33	♂	44.4	Diazepam syrup 8 mL po	MMK Restraint and direct injection	**Ketamine (10mg/mL) CRI**: 10 mL/h → 12 mL/h → 15 mL/h → 20 mL/h **Propofol bolus + CRI**:100 mg bolus(150 mL/h) → 50 mL/h → 25 mL/h
Ai GAIN# 0434	40	♀	56.9	NA	MMK direct injection by TM	**Propofol bolus + CRI**: 120 mg bolus (150 mL/h) → 23.2 mL/h

**Table 2:** Attempt of human propofol TCI in chimpanzees (2016–2018). MMK: Medetomidine 0.012 mg/kg+Midazolam 0.12 mg/kg +Ketamine 3.5 mg/kg

Name GAIN#	Age (at anesthesia)	Sex	Body weight (kg)	Pre-med	Induction	Maintenance
Pan GAIN# 0440	33	♀	53.8	NA	MMK Telinject	Propofol TCI Target: 2 μg/mL → 3 μg/mL
Akira GAIN# 0435	41	♂	60.1	NA	MMK Telinject	Propofol TCI Target 2 μg/mL
Pendesa^18, 19^ GAIN# 0095	40	♀	50.8	NA	MMK Training and squeezing cage	Propofol TCI Target 2 μg/mL
Ayumu GAIN# 0608	17	♂	60.5	NA	MMK Telinject	Propofol TCI Target 2 μg/mL
Gon GAIN# 0437	52	♂	64	NA	MMK Direct injection by NM	Propofol TCI Target 2 μg/mL
Popo 0438 GAIN#	35	♀	50.6	NA	MMK Telinject	Propofol TCI Target: 2 μg/mL → 3 μg/mL

### Predictive performance of human pharmacokinetic models

**Figure 1:**
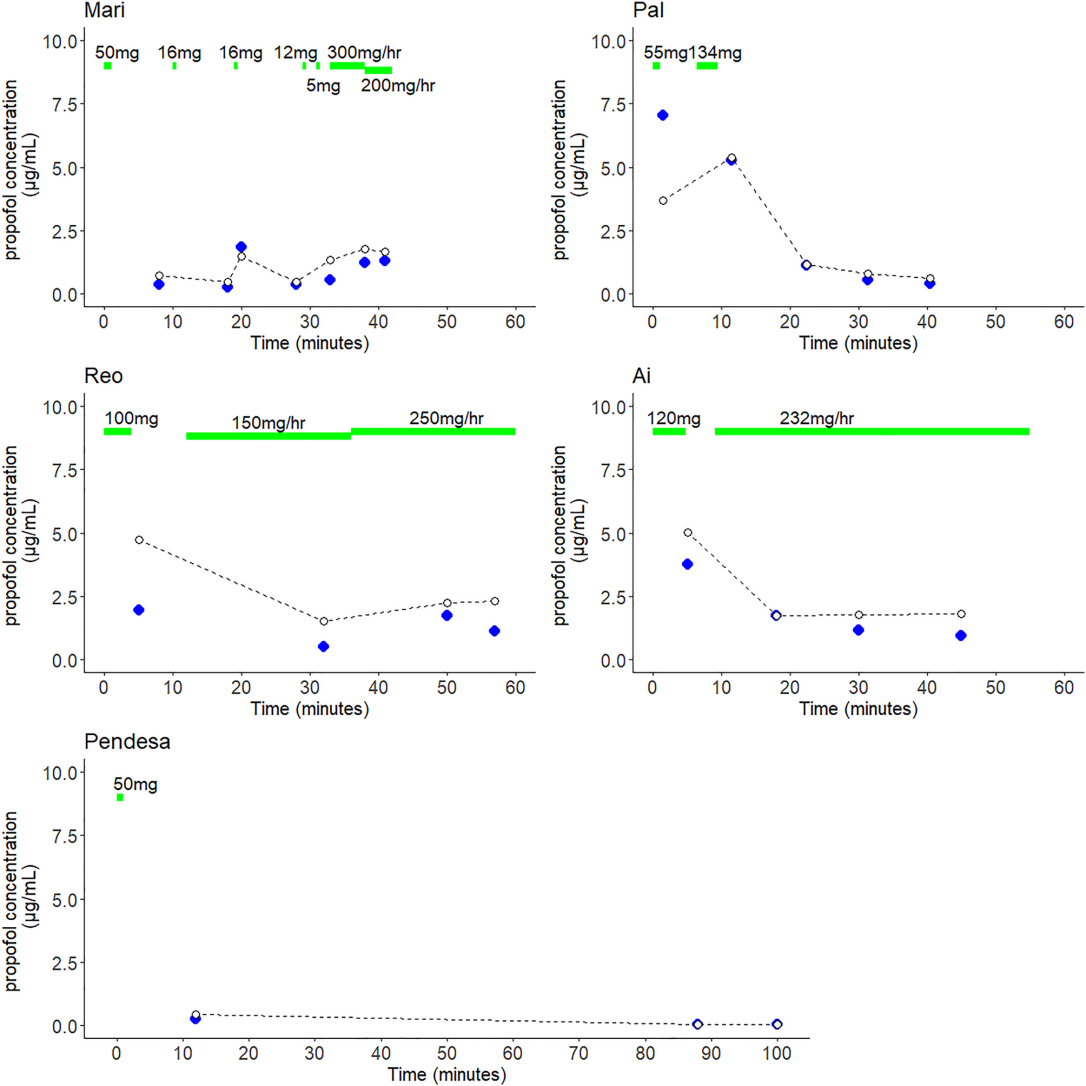
Time course of Cps following propofol bolus and/or infusion in five chimpanzees. Blue circles represent measured Cps and white circles and dotted lines indicate predicted Cps using Marsh model. Green bars indicate amount of propofol administration.

Figure 1 shows the time course of propofol administration along with the measured concentrations and the predicted plasma concentration (Cp) for each chimpanzee using the Marsh model. Supplemental Figures 1–3 show the plasma concentrations predicted by the Schnider, Eleveld volunteer and Eleveld PKPD models, respectively. For the five chimpanzees, median performance error (*Median PE*) and median absolute performance error (*Median APE*)^21^ for Marsh, Schnider and Eleveld volunteer and Eleveld PKPD models were − 31% and 33%, − 13% and 27%, 10% and 32%, 9% and 23%, respectively.

As seen in Fig. 1, measured and predicted concentrations were similar for most of the time. The difference deviated more in the early phase compared to later phases (Pal). For Reo and Ai, the measured concentrations were lower but the changes were parallel to the predicted concentrations.

### Human TCI in chimpanzees

Propofol TCI using human TCI pump was performed in six chimpanzees. Propofol TCI was performed for 76 ± 19 min. At termination of the propofol TCI, atipamezole was administered to reverse the effects of medetomidine given for induction of anesthesia. The chimpanzees started to move spontaneously at 11 ± 5 min after termination of propofol TCI and started to sit at 26 ± 14 min except for Pendesa who lay down for more than 1 h on both occasions.

**Figure 2:**
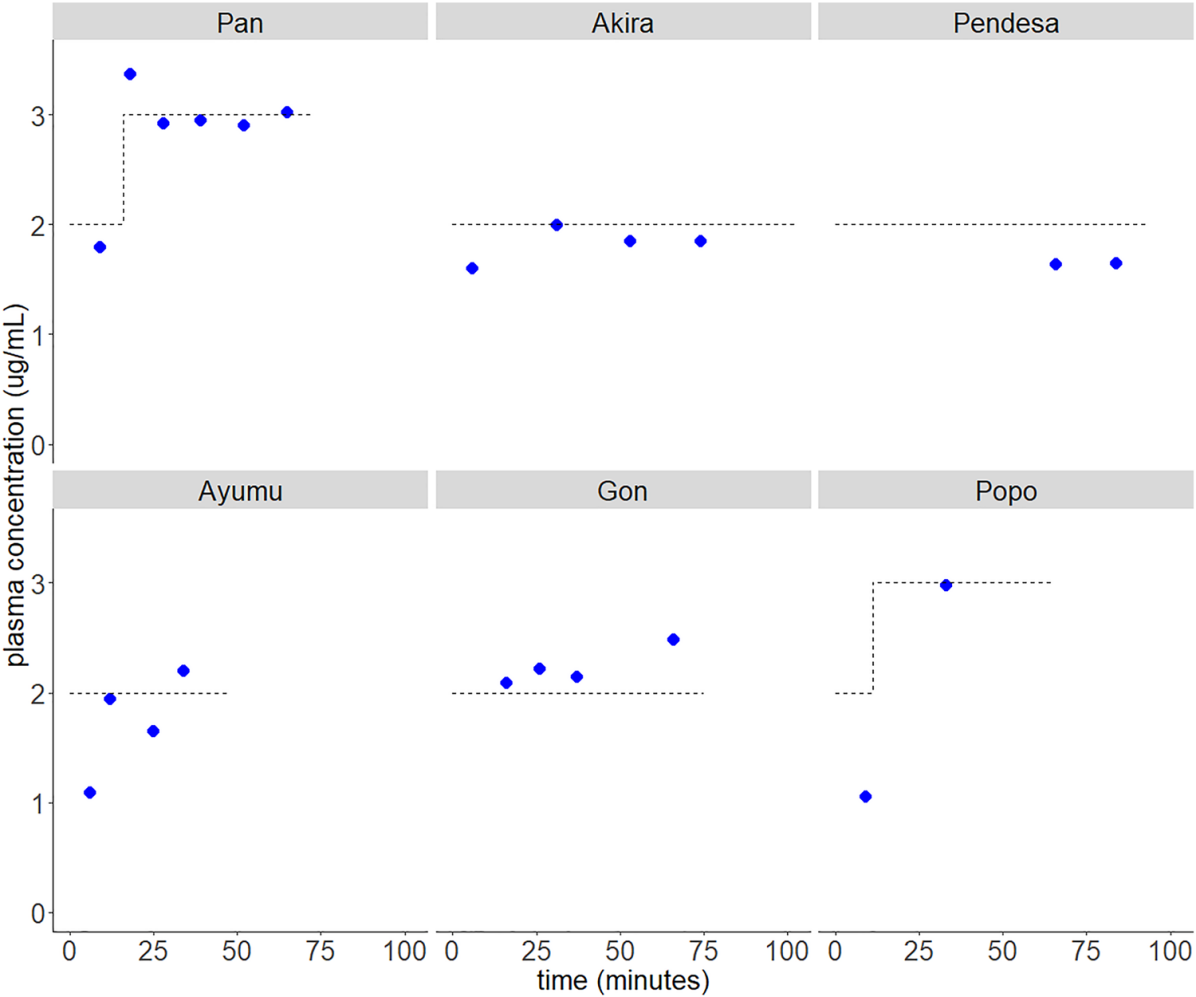
Time course of Cps following propofol TCI in six chimpanzees. A TCI pump programmed with Marsh model was used. Blue circles represent measured Cps and dotted lines indicate target Cps in TCI.

Figure 2 shows the time course of target Cp and measured Cp in each individual. The measured Cp was close to the target Cp for most of the time for the most individuals. The measured Cp deviated more in early phase (e.g. Akira 6 min, Ayumu 5 min, and Popo 9 min) but was closer to the target Cp in the later phases.

**Figure 3:**
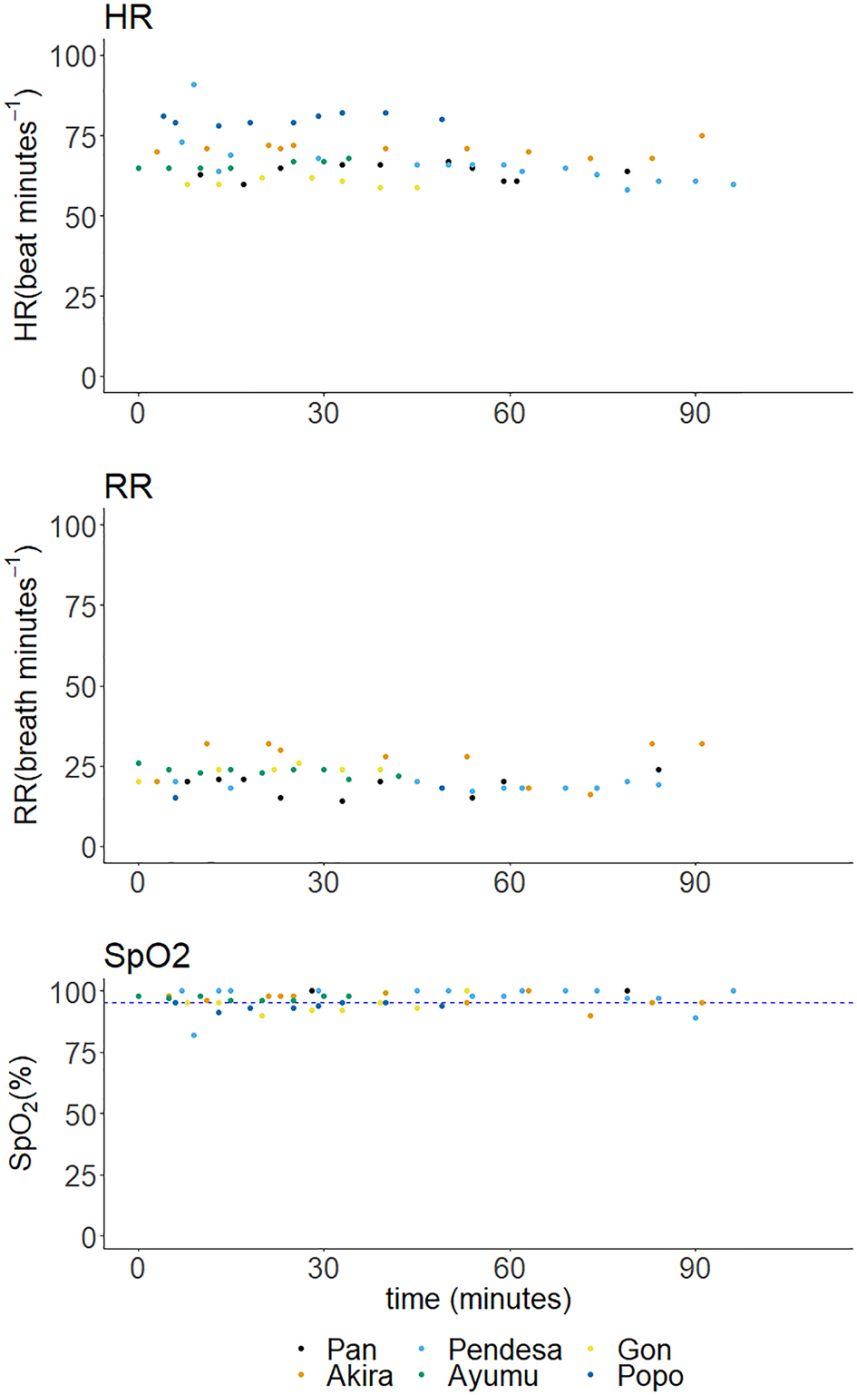
Time course of heart rate (HR), respiratory rate (RR) and percutaneous arterial blood oxygen saturation (SpO_2_) following propofol TCI in six chimpanzees. Each colored dots indicate values in each individual.

*Median PE* and *Median APE* were − 9% and 13% respectively. Figure 3 shows the time course of HR, RR and SpO_2_ during propofol TCI. There were individual differences, but HR and RR were stable within an individual. Apnea was not seen except in Pendesa who has an arachnoid cyst^18, 19^ and had had apneas on several previous occasions when anesthesia was maintained with sevoflurane. For the current anesthetic, a planned endotracheal intubation was performed, and when apnea was seen, she was manually ventilated for several minutes and dimorpholamine was administered, following which spontaneous breathing recovered.

In all six individuals, recovery was rapid and smooth. Ayumu showed marked disinhibition during recovery. He screamed and displayed within 30 min of propofol termination. SpO_2_ was above 95% for most of the time. Decreases of SpO_2_ were seen in Pendesa following apnea but soon recovered to above 95%.

## Discussion

In this study, we first investigated whether plasma propofol concentrations in chimpanzees are adequately predicted using human pharmacokinetic models following manual bolus and infusion administration of propofol. *Median PE* and *Median APE*, the indices of bias and accuracy, were within acceptable range for the Schnider, Eleveld volunteer and Eleveld PKPD modes and were close to the acceptable range for the Marsh model. The difference between measured and predicted Cps deviated more in the early phase compared to later phases. This observation is consistent with previous reports that the measured propofol Cps can differ greatly from predicted Cps during the first few minutes of administration in humans^22^ and also in Japanese macaques^23, 24^ and common marmosets^25^. This is probably because these models make the incorrect assumption of immediate mixing of administered drugs within the central compartment. Our second question was whether we could apply TCI administration of propofol in chimpanzees, using a human TCI pump. Propofol TCI using Marsh TCI pump was attempted in six chimpanzees. *Median PE* and *Median APE* were within the clinically acceptable range.

The results suggest that propofol anesthesia using a TCI pump is a good alternative to inhalation anesthesia in chimpanzees. Cardiorespiratory parameters (HR and RR) were stable during propofol TCI. The operation of the pump was easy, and the anesthesia was very stable in all individuals. The advantages of propofol intravenous anesthesia include but are not limited to (1) no risks of environmental pollution with volatile anesthetics and thus no exposure of the personnel, (2) a syringe pump is light weight and can more easily be taken to the housing environment compared to an anesthetic machine including circuits and vaporizers, (3) a syringe pump is also easier to be transported with a chimpanzee when it is necessary to transport an anesthetized chimpanzee, e.g. from treatment room to recovery room or from an old facility to a new facility. In addition, it is also expected that propofol intravenous anesthesia causes less nausea and vomiting as in humans. Further studies are required to verify the incidence of nausea and vomiting in chimpanzees.

In this study, apnea was not seen except in Pendesa who had history of having apnea during anesthesia. In KUPRI, chimpanzees are not routinely intubated unless there are increased risks, because (1) Masks for humans fit chimpanzees well and it is possible to ventilate using masks, (2) chimpanzees often cough after extubation, (3) intubation itself can induce an apnea reflex. Nevertheless, it is important to prepare for intubation as well as for bag-valve-mask ventilation in case of prolonged apnea.

One of the limitations of this study is that the number of chimpanzees anesthetized was small. Recently, in KUPRI we have had 3 to 4 opportunities to anesthetize chimpanzees for regular veterinary examinations per year. In human medicine, approximately 2 million individuals undergo general anesthesia per year in Japan^26^. In one hospital in the Netherland (University Medical Center Groningen), more than 10, 000 patients receive one or more drugs by TCI per annum and it was estimated that TCI is used in 2.6 million patients in Europe and approximately 5 million patients in the world^8^. The Marsh model^9^ was based on an evaluation of the pharmacokinetics of propofol in 18 patients and Schnider model^10^ was based on data from 24 healthy volunteers. In contrast, Eleveld and colleagues^11, 12^ used data from multiple institutions; more than 15,000 observations from more than 1000 individuals with an age range from 0 to 88 year, and a weight range from 0.68 to 160 kg. Furthermore the Eleveld model uses allometric scaling of clearance values, and this might theoretically result in the model extrapolating more accurately across species, while Marsh and Schnider model do not use allometric scaling. Although it would not be possible to collect such large scale data in chimpanzees, further collection of data from multiple collaborating institutions may elucidate problems and applicability to various cases.

When the TCI pump is not available, it is also possible to use human PK models to perform simulations to plan the dose regimen for manual infusion of propofol in chimpanzees using a conventional syringe pump. A step-down infusion, in which the infusion rate is decreased stepwise in order to maintain a desirable plasma concentration, was used as an alternative method to computer-controlled infusion before TCI was accepted and widely used in humans^27^. In macaques, a step-down infusion of propofol based on simulations of the dose regimen was feasible and stable plasma concentration of propofol during continuous infusion was achieved^24^.

When using propofol TCI or manual infusion for invasive procedures, it is necessary to administer adequate doses of analgesics, since propofol does not have analgesic effects. Balanced anesthesia is well recognized in both human and veterinary medicine. In human medicine, TCI for opioid analgesics including remifentanil, sufentanil, alfentanil are also available using a commercially available PK pump (e.g. Alaris PK anaesthesia pump). Further research will elucidate the possibility of opioid TCI in chimpanzees. In addition, it is also possible to perform simulations of dose regimen for opioid analgesics and adapt them in chimpanzees as a guide.

Chimpanzees have history of being used as models for humans as their body weight, genetical background and physiology are similar to that of humans^28, 29^, which suggests that the human models are likely to be acceptable for use in chimpanzees. Our results suggested that it is actually the case for propofol pharmacokinetic models. There are collaborative efforts between human medical professionals (doctors and dentists) and veterinarians including the great ape heart project^30^ and chimpanzee dental project (collaboration between KUPRI and Tsurumi University, e.g.^31^). The collaboration and “One Health approach”^32^ are particularly fruitful in conservation of endangered great ape species in captivity. In this study, the collaboration between human anesthesiologists and veterinarians made it possible to use human propofol TCI in chimpanzees. Although there are no commercially available TCI pumps for nonhuman animals and TCI is only performed at research level in dogs^21, 33^, veterinarians can easily learn and use TCI pump to anesthetize chimpanzees with help from human doctors.

## Conclusion

It would be clinically acceptable to use human pharmacokinetic models for propofol administration in chimpanzees and it is feasible to use a human TCI pump for this purpose.

## Methods

### Animals

Ten chimpanzees (seven females and three males) were anesthetized for regular veterinary examination from 2014 to 2018. The demographic data is listed along with anesthetic protocols in Tables 1 and 2. A chimpanzee named Pendesa had two occasions for regular veterinary examinations during the period. Anesthesia was induced with intramuscular administration of the combination of medetomidine 0.012 mg/kg, midazolam 0.12 mg/kg and ketamine 3.5 mg/kg (MMK) with or without oral premedication using diazepam or midazolam. Propofol was administered as a constant rate infusion, as an “induction” agent and/or adjunctive agent along with sevoflurane inhalation or ketamine infusion from 2014 to 2016 (Table 1). Propofol TCI using a human TCI pump (TE-371, Terumo Corporation., Tokyo) was attempted from 2016 to 2018 (Table 2). Intravenous catheters were placed into the cephalic vein of each arm, one for propofol administration, and the other for blood sampling. Blood samples were taken 3–7 times following propofol administration. Vital signs including heart rate (HR), respiratory rate (RR), rectal temperature (RT) and oxygen saturation (SpO_2_) were monitored using a multi-parameter anesthetic monitor BP-608 Evolution (Omron-Collin, Tokyo), iSpO_2_ pulse oximeter (Masimo Japan, Tokyo). If RR was not detected, the respiratory movements of the thorax were counted.

### Measuring the plasma concentration

The plasma concentration (Cp) of Propofol was determined by high performance liquid chromatography (HPLC) using a fluorescence detector at 310 nm after excitation at 276 nm (RF-550, CTO-10AS, LC-10AD, SIL-10AD, SCL-10A, and DGU-14A; Shimadsu, Kyoto, Japan). The protocol for measurement was described in a previous study^22^. Briefly, the mobile phase was acetonitrile–water-phosphoric acid (55:45:2 by volume) at a flow rate of 1.0 ml min^−1^. The column temperature was 30 °C. Thymol was used as an internal standard. To remove proteins prior to injection the plasma samples were prepared with a solid-phase extraction.

### Pharmacokinetic model evaluation

Four human PK models; the Marsh^9^, Schnider^10^ and Eleveld volunteer^12^ and Eleveld PKPD models^11^, were used to predict Cp for each case using NONMEM software version 7.3 (ICON plc, Dublin, Ireland). Figures were created using the ggplot2 package^34^ in R statistical software v. 3.3.3^35^. Predictive performance of the different models was assessed with the Varvel criteria median performance error (*Median PE*) and median absolute performance error (*Median APE*) as the following equations^36^:

$$PE=\frac{measuredCp-predictedCp}{predictedCp}\times 100$$

$$APE=|PE|$$

$${MedianPE}_{i}=median\left\{{PE}_{ij},j=1,\dots ,{N}_{i}\right\}$$

$${MedianAPE}_{i}=median\left\{{|PE}_{ij}|,j=1,\dots ,{N}_{i}\right\}$$

Clinically acceptable range was considered as − 20% < Median PE < 20%, and Median APE < 30%^37^.

### Human TCI in chimpanzees

A human TCI pump programmed with the Marsh model was used for six chimpanzees. Only Marsh model TCI pumps were commercially available in Japan. Target concentration was initially set at 2 μg/mL and increased to 3 μg/mL if necessary.

*Median PE* and *Median APE* were calculated to evaluate the performance of human TCI in chimpanzees as mentioned above, except that the target Cp, which is calculated using PK models as predicted Cp, was used instead of predicted Cp. The quality of recovery (ex., smooth recovery, agitation, over-excitement, vocalization) was observed and adverse effect including coughing, nausea and vomiting were recorded as they occurred.

### Ethical approval

This study was conducted under the Guidelines for Care and Use of Nonhuman Primates (Version3) provided by the Primate Research Institute, Kyoto University (KUPRI). Animal Welfare and Care Committee at KUPRI approved the protocols (Protocol Numbers: 2014-103, 2015-092, 2016-116, 2017-095, 2018-067), and then the protocols were authorized by the Kyoto University Animal Experimentation Committee.

## Supplementary Information

Supplementary Figure S1.

Supplementary Figure S2.

Supplementary Figure S3.

Supplementary legends.
