# Supplementary figures and images for: Propofol infusions using a human target controlled infusion (TCI) pump in chimpanzees (Pan troglodytes)
Source: Sci Rep. 2021 Jan 13;11:1214. doi: 10.1038/s41598-020-79914-7 (PMC7806914; doi:10.1038/s41598-020-79914-7)

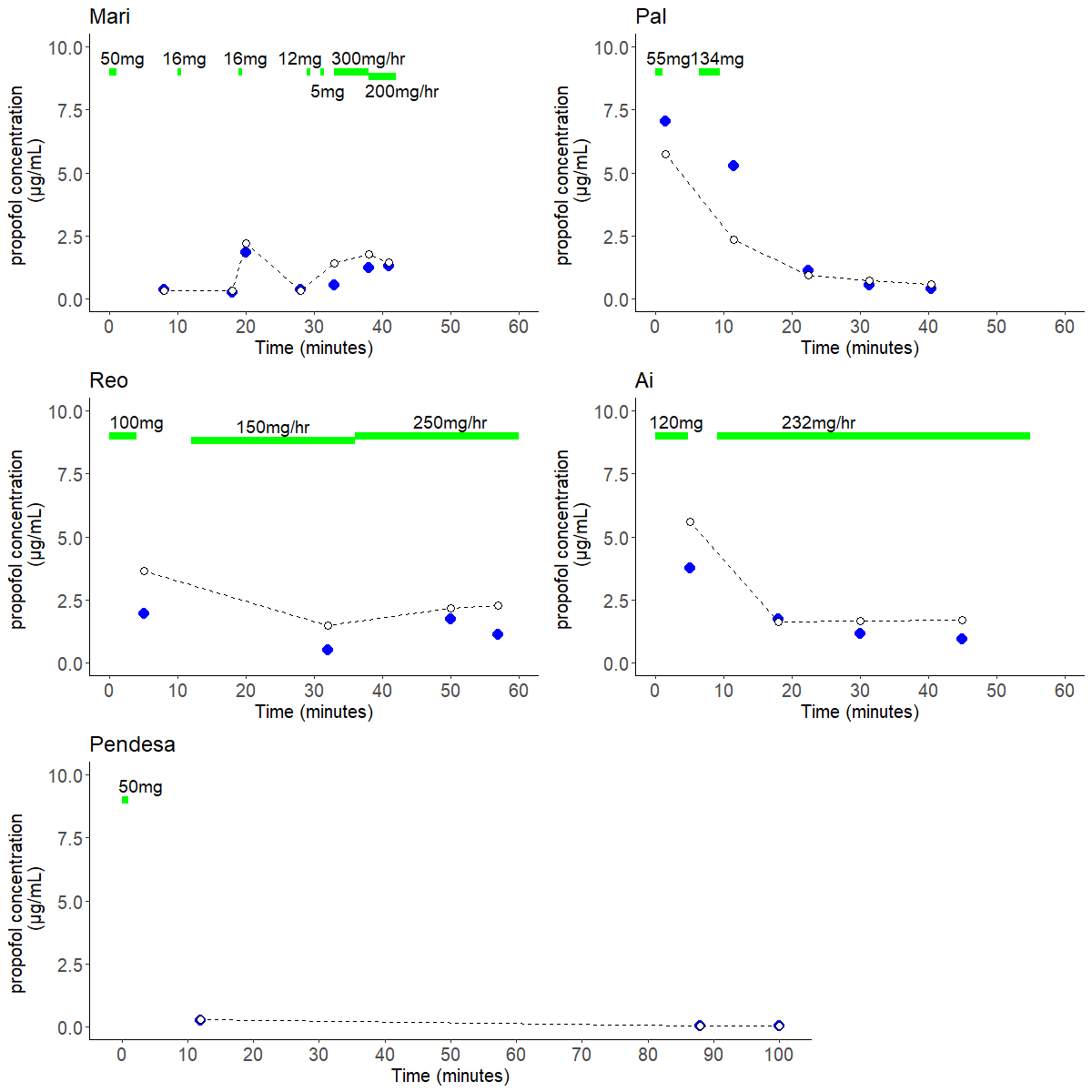

Supplement: Supplementary file 1 — Supplementary Figure S1. [file 41598_2020_79914_MOESM1_ESM.tiff]

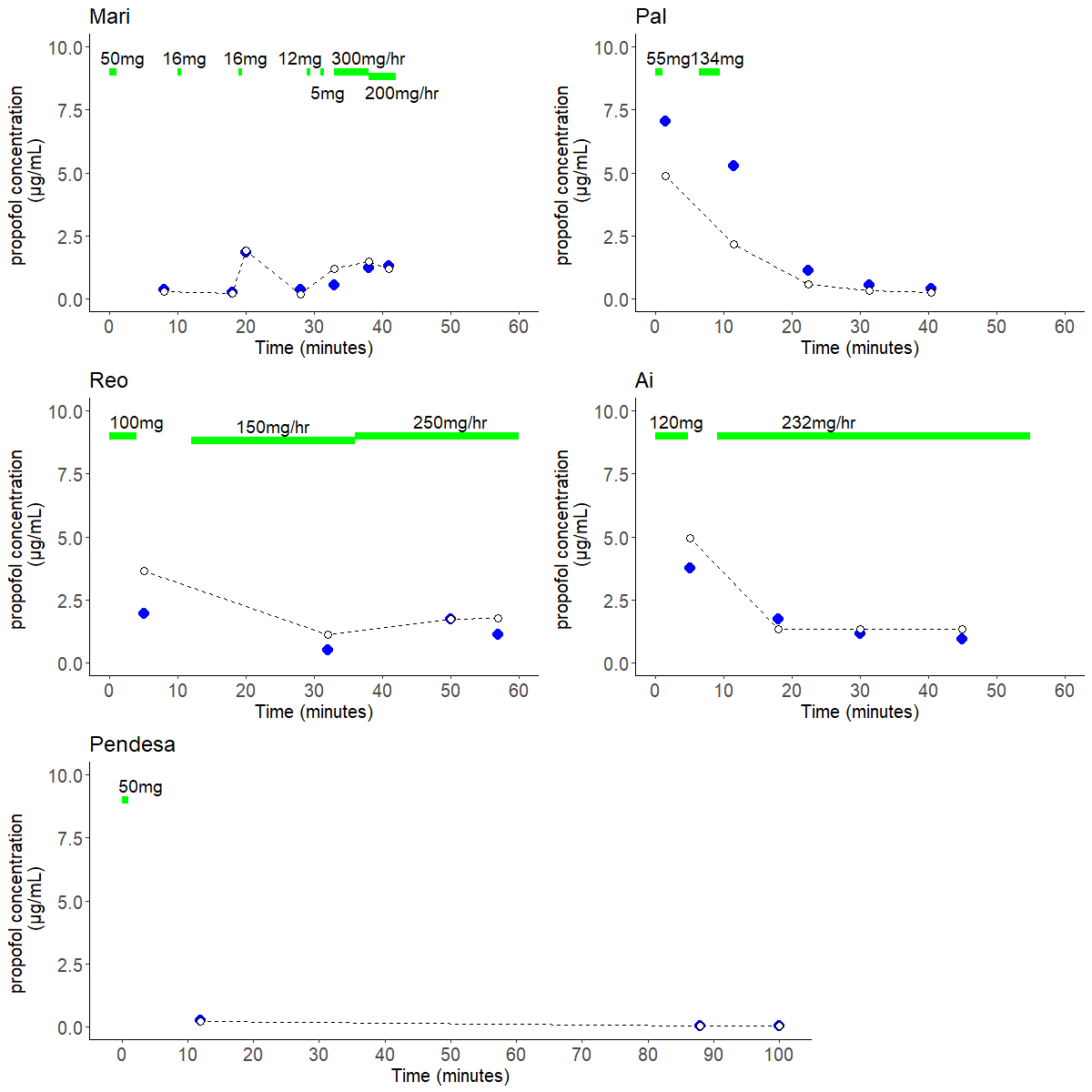

Supplement: Supplementary file 2 — Supplementary Figure S2. [file 41598_2020_79914_MOESM2_ESM.tiff]

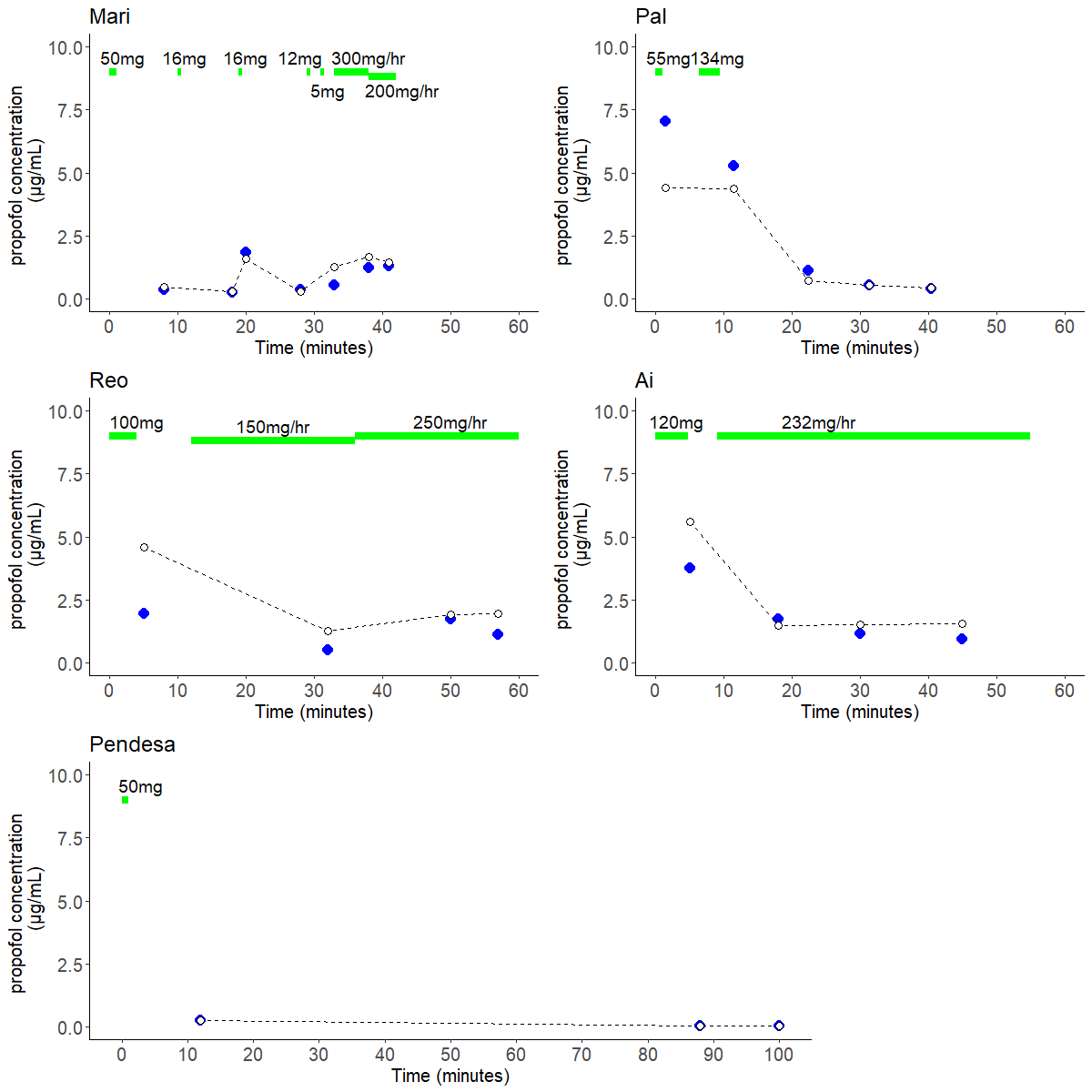

Supplement: Supplementary file 3 — Supplementary Figure S3. [file 41598_2020_79914_MOESM3_ESM.tiff]
