# Supplementary material for: Propofol infusions using a human target controlled infusion (TCI) pump in chimpanzees (Pan troglodytes)
Source: Sci Rep. 2021 Jan 13;11:1214. doi: 10.1038/s41598-020-79914-7 (PMC7806914; doi:10.1038/s41598-020-79914-7)
Supplement: Supplementary file 4 — Supplementary legends. [file 41598_2020_79914_MOESM4_ESM.docx]

Propofol infusions using a human target controlled infusion (TCI) pump in chimpanzees (*Pan troglodytes*)

Miyabe-Nishiwaki T^1*^, Kaneko A^1^, Yamanaka A^1^, Maeda N^1^, Suzuki J^1^, Tomonaga M^1^, Matsuzawa T^2^, Muta K^3^, Nishimura R^3^, Yajima I^4^, Eleveld DJ^5^, Absalom AR^5^, Masui K^6*^

1. Primate Research Institute, Kyoto University, Inuyama, Aichi, Japan

2. Kyoto University Institute for Advanced Study, Kyoto, Japan

3. Laboratory of Veterinary Surgery, Graduate School of Agricultural and Life Sciences, the University of Tokyo, Bunkyo-ku, Tokyo, Japan

4. Department of Pharmacy, National Defense Medical College Hospital, Tokorozawa, Saitama, Japan

5. University Medical Center Groningen, Groningen, Netherland

6. Department of Anesthesiology, Showa University School of Medicine, Shinagawa-ku, Tokyo, Japan

Corresponding to

Kenichi Masui

Email: kenichi@masuinet.com

Takako Miyabe-Nishiwaki

Email: miyabe.takako.2s@kyoto-u.ac.jp

**Figure S1** Time course of Cps following propofol bolus and/or infusion in five chimpanzees. Blue circles represent measured Cps and white circles and dotted lines indicate predicted Cps using Schnider model. Green bars indicate amount of propofol administration.

**Figure S2** Time course of Cps following propofol bolus and/or infusion in five chimpanzees. Blue circles represent measured Cps and white circles and dotted lines indicate predicted Cps using Eleveld volunteer model. Green bars indicate amount of propofol administration.

**Figure S3** Time course of Cps following propofol bolus and/or infusion in five chimpanzees. Blue circles represent measured Cps and white circles and dotted lines indicate predicted Cps using Eleveld PKPD model. Green bars indicate amount of propofol administration.
